# Total rhinectomy with prosthesis placement as a treatment for moderately differentiated squamous cell carcinoma of the nose

**DOI:** 10.1002/ccr3.6957

**Published:** 2023-02-13

**Authors:** César Gamaliel Rivera Martínez, Edgar Hernández Abarca, José Pablo Busto Ruano, Mayrelle Martínez Quincosa, Raymundo Benjamín Priego Blancas, Adelaido López Chavira, Jorge Alberto Romo Magdaleno

**Affiliations:** ^1^ Department of Otorhinolaryngology Head and Neck Surgery Central Military Hospital Mexico City Mexico; ^2^ Instituto Tecnológico de Estudios Superiores de Monterrey Mexico City Mexico; ^3^ Department of Plastic and Reconstructive Surgery Centro Médico ABC Mexico City Mexico; ^4^ Department of Otorhinolaryngology Head and Neck Surgery Corporativo Hospital Satélite Mexico City Mexico

**Keywords:** nasal carcinoma, prosthesis, total rhinectomy

## Abstract

Squamous cell carcinoma is one of the most common head and neck types of skin cancer. This main objective of this paper is to present a case of a patient who had a moderately differentiated squamous cell carcinoma of the nose and whose tumor had an aggressive growth.

## Graphical abstract

Patients with invasive nasal squamous cell carcinoma who require total rhinectomy and adjuvant chemotherapy benefit from a self‐retaining nasal prosthesis, as it promises favorable cosmetic and functional results and decreased tumor recurrence.

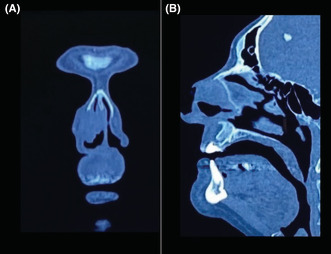

## 1 INTRODUCTION

The nose is a typical subsite of head and neck skin cancers, being the squamous cell carcinoma the most common histological subtype.^1^, ^2^, ^3^ This type of tumor shows a locally aggressive growth and tends to invade cartilage and deeper structures requiring a subtotal or total rhinectomy when they are larger than 1 cm and have greater invasion of the surrounding structures.^1^, ^2^, ^3^ Although total rhinectomy leads to excellent tumor control, it alters the patient's appearance, disturbs facial harmony, and may affect nasal function depending on the resected elements.

Also, the majority of these patients require radiotherapy which can affect the quality of the bone and tissue. In these cases, it is difficult to reconstruct the nose aesthetically and functionally with skin flaps.^1^ For this reason, an excellent alternative is a nasal prosthesis which is safe and implies better oncological control and limits tumor recurrences. This device also mimics the original appearance and function of the nose prior to surgery, thereby improving the patient's quality of life.^2^

The main objective of this manuscript is to present a case of a patient with moderately differentiated squamous cell carcinoma of the nose, who underwent total rhinectomy and posterior reconstruction with nasal self‐ retained prosthesis.

## 2 CASE REPORT

**FIGURE 1:**
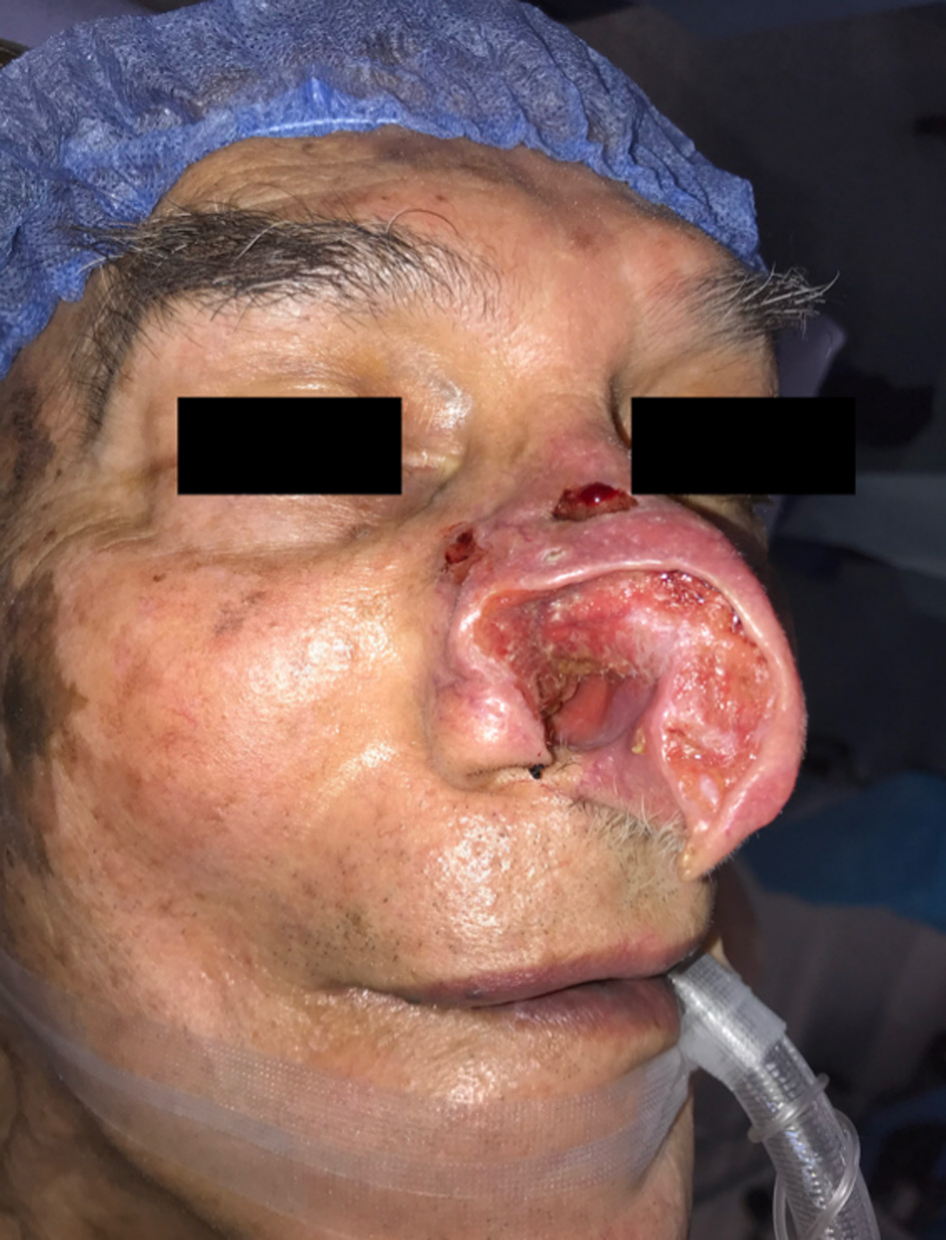
Patient with nasal tumor

An 88‐year‐old patient with a history of epidermoid carcinoma on the left cheek skin resected 12 years ago. He had a lesion in the right nasal wing with full‐thickness perforation and the presence of an obstructing tumor in the entire right nostril, invading the septum for the last 2 years (Figure 1).

**FIGURE 2:**
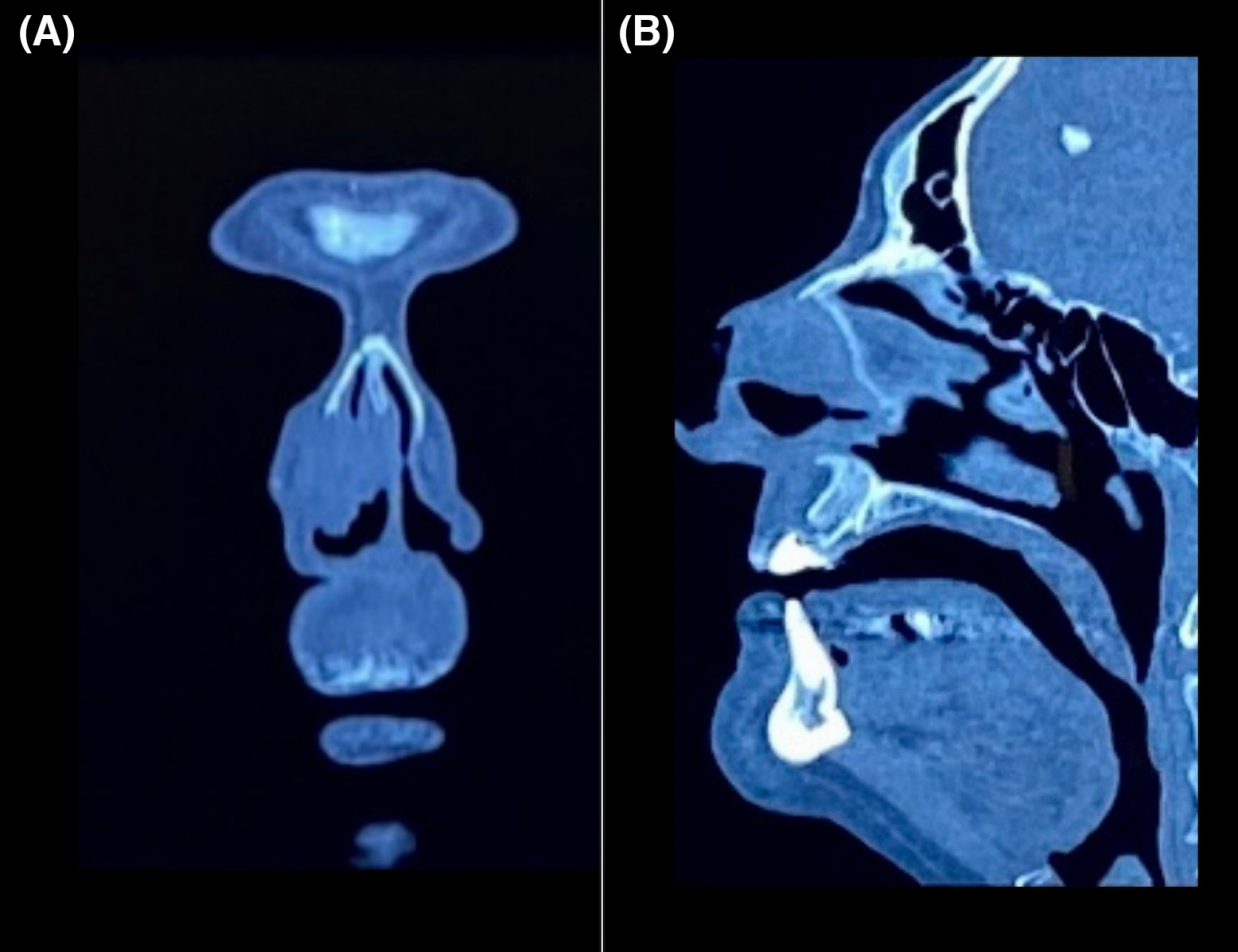
(A) Bone window tomography on coronal section: right nostril is occupied on 80% by a tumor that is in intimate contact with the septum on its cartilaginous portion with no bone erosion. (B) Bone window CT on sagittal section: tumor is seen in the right nostril in contact with the cartilaginous septum, no bone erosion is observed and the nasal bones are spared. No involvement of the paranasal sinuses and skull

Not contrasted CT reported tumor invasion of the nasal wing, septum, right nasal dorsum, right inferior turbinate in its frontal area; it did not involve nasal floor, maxilla or paranasal sinuses (Figure 2). It was staged as T3N0M0 according to AJCC eighth edition of primary cutaneous carcinoma. A biopsy was taken with histopathological results of moderately differentiated squamous cell carcinoma.

**FIGURE 3:**
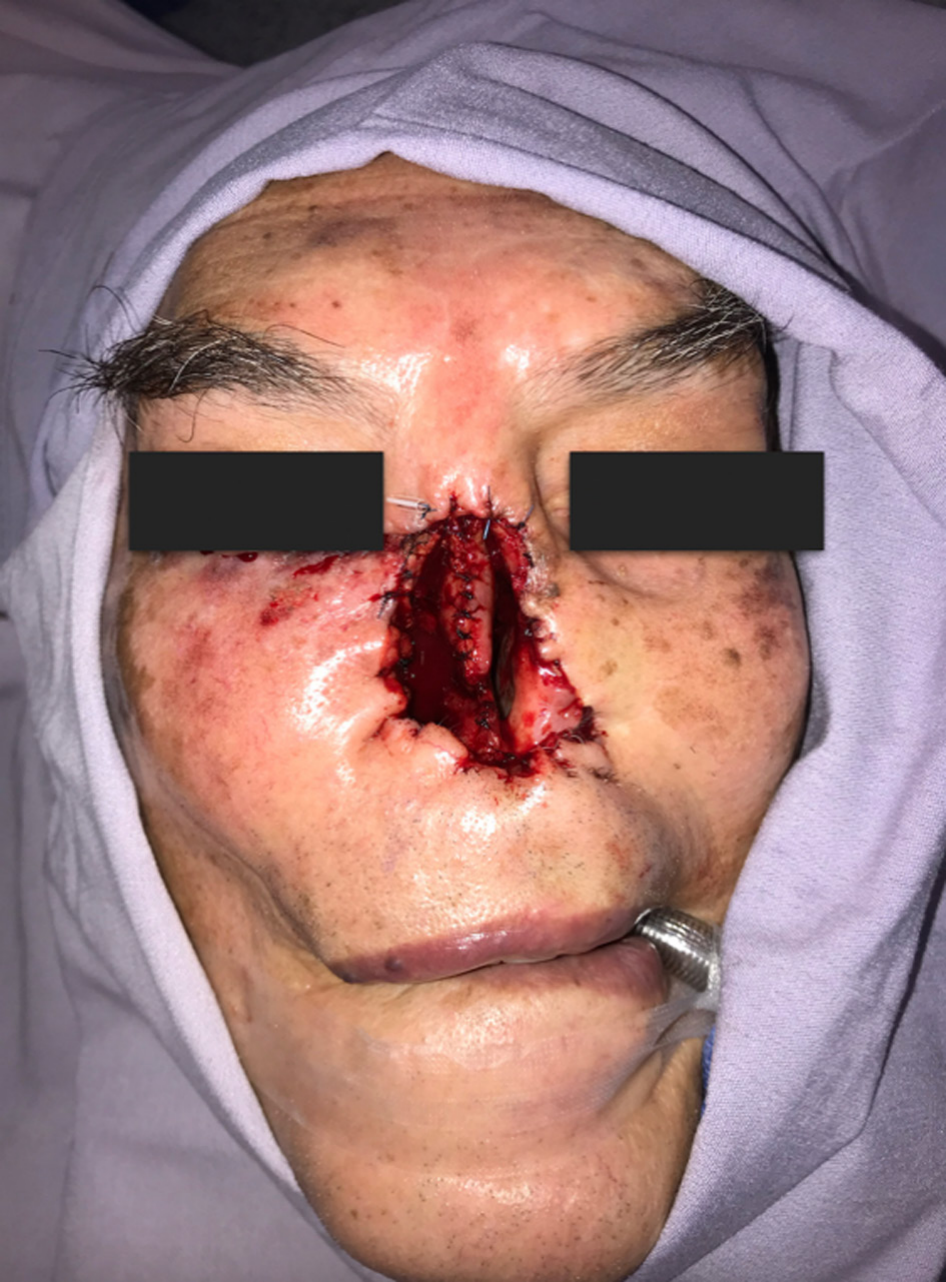
Patient with nasal defect after total rhinectomy

Total rhinectomy was scheduled, which was performed without complications, obtaining a pathology result that confirmed moderately differentiated, invasive and ulcerated squamous cell carcinoma of 3.3 × 2.3 cm deep that invaded the nasal mucosa up to the elastic cartilage of the nasal septum and negative skin edges for neoplasia (T3N0M0; Figure 3). The case was evaluated by the tumor board in which otorhinolaryngology, radiology, and oncology departments participated in order to discuss the proper treatment according to the patient's age, health situation and cancer stage. Therefore, the patient received adjuvant radiotherapy (45 Gy in 15 sessions) followed with no complications nor recurrence due to clinical manifestations or PET‐CT during 1 year follow‐up .

Total rhinectomy was scheduled and performed as follows: Surgical marking of the affected area was done, leaving 1 cm tumor‐free macroscopic margin. The margin of the posterior septum was delimited, endoscopically assisted. A complete en bloc resection was performed, subsequently a mustarde advancement flap was made to cover the defect on the lateral skin of the nasal pyramid, and the rest of the healthy tissue was fixed to the pyriform aperture (Figure 3).

**FIGURE 4:**
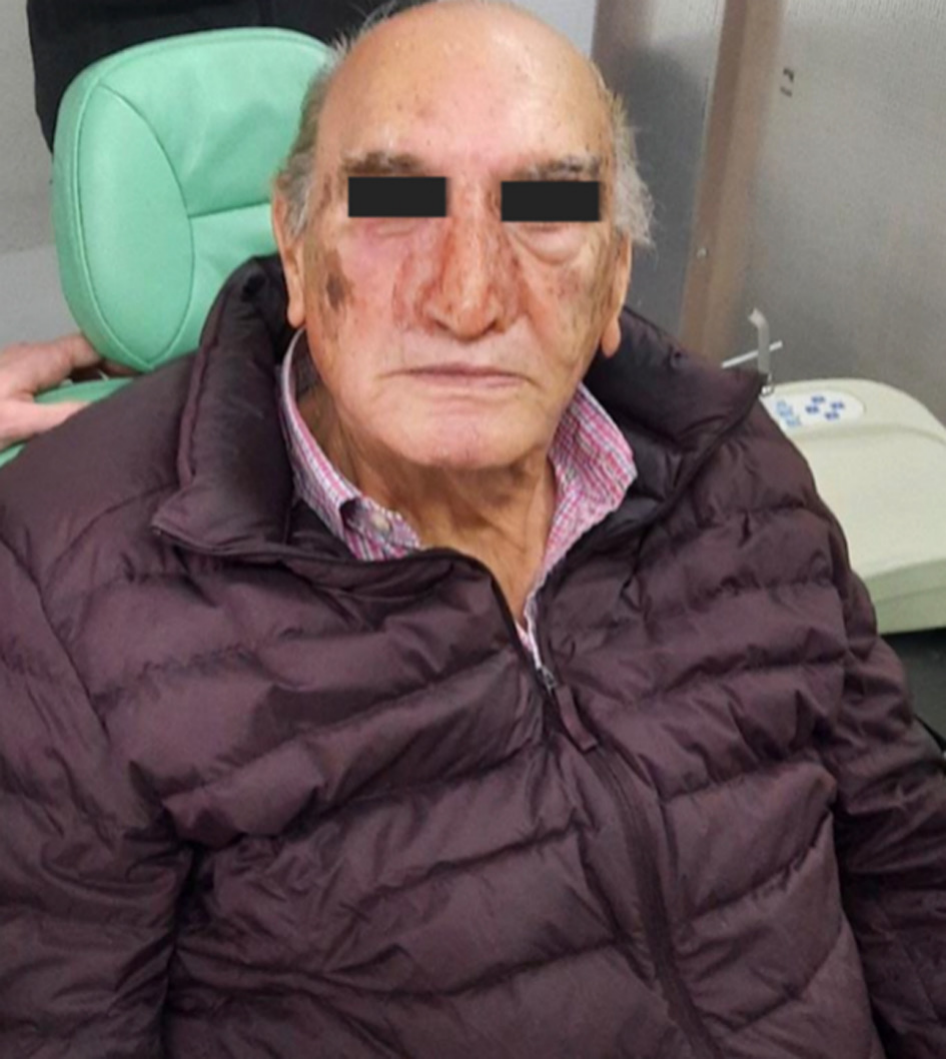
Patient after total rhinectomy with implant‐retained prosthesis

4.5 months after the rhinectomy, the patient showed healing of the skin and mucosa of the nasal defect. Therefore, the reconstructive surgery department evaluated a nasal prosthesis placement. Maxillofacial department was in charge of the prosthesis impression. The implant‐ retained prosthesis was made out of silicone, mimicked the natural skin of the patient, and was chemically attached (Figure 4).

The patient was followed up by the tumor board, who agreed to perform a PET‐CT one year after the surgical intervention, no recurrence was identified. In addition, during the first year, the patient was seen every 3 months for physical evaluation, ultrasound and chest X‐ray with no evidence of recurrence.

## 3 DISCUSSION

In this case, the patient underwent a total rhinectomy which was preferred based on the histological type, and the rapid growth, which led to better tumor control. No immediate adverse effects were present during the rhinectomy procedure nor the prosthesis placement.

The overall advantages of a total rhinectomy with prosthesis placement were the lack of donor site morbidity and graft rejection, shorter surgical time compared to procedures involving graft placement, better aesthetic appearance since the color and the texture of the prosthesis to that of patient's skin prior to the surgical procedure was assimilated, while maintaining good functionality of the nose. Although rhinectomy promises a lower risk of recurrence and good tumor control, it is important to mention the adverse effects, which can be either local symptoms such as numbness, swelling and tightness of the face as well as generalized symptoms such as headache, lightheaded, and burning sensation, which did not occur in this patient.^2^, ^4^

Tissue invasion by this tumor required surgery which causes severe facial deformity and decreased nasal function. Unlike nasal reconstructions with autologous flaps, the type of prosthesis used does not require subsequent surgical approaches and is not affected by bone and tissue damage after adjuvant radiotherapy.^4^

In addition, it improves the quality of life as it gives a good functional and aesthetic result. By having a self‐ retained prosthesis with similarity to the natural nose prior to surgery, it increases the patient's self‐esteem and social activity. It is important for the patient to go to therapy or get used to the prosthesis because functionality might get altered. Although it is recovered, it can cause difficulty in communication, breathing, swallowing, eating, and drinking.^2^ In this case, the patient attended three rehabilitation sessions in which he was taught how to maintain a proper cleaning of the prosthesis and how to take care of the skin around it. Also, exercises were performed in order to improve the patient's phonetics. No further rehabilitation was required since the patient did not present discomfort, breathing difficulties and maintained normal function. However, the patient was granted to freely come on attending to the rehabilitation center to solve any doubt or report discomfort.

## 4 CONCLUSION

Squamous cell carcinoma is the most common histological subtype of tumor of the head and neck. The patient presented a moderately differentiated squamous cell carcinoma of the nose which invaded the nasal wing, septum, right nasal dorsum, and right inferior turbinate in its frontal area. Therefore requiring a total rhinectomy and adjuvant radiotherapy as treatment, no complications were reported.

Total rhinectomy not only involves facial deformity, but functional and psychological impairments are also seen. Adjuvant radiotherapy causes bone and tissue damage, which is why chemically self‐retained nasal prosthesis is the treatment of choice, as it decreases tumor recurrence, does not require subsequent surgical approaches, and improves both nasal function and facial aesthetics by matching the shape and nasal skin of the patient prior to the condition.

## AUTHOR CONTRIBUTIONS

**Cesar Gamaliel Rivera Martinez:** Resources; validation. **Edgar Hernandez Abarca:** Investigation; resources; supervision. **José Pablo Busto Ruano:** Conceptualization; formal analysis; resources; supervision; validation. **Mayrelle Martinez Quincosa:** Conceptualization; formal analysis; investigation; resources; writing – original draft; writing – review and editing. **Raymundo Benjamín Priego Blancas:** Supervision; validation. **Adelaido López Chavira:** Supervision; validation; visualization; writing – review and editing. **Jorge Alberto Romo Magdaleno:** Supervision; validation; visualization; writing – review and editing.

## FUNDING STATEMENT

This research received no specific grant from any funding agency in the public, commercial, or not‐for‐profit sectors.

## CONFLICT OF INTEREST

The authors of this manuscript certify that they have no affiliations with or involvement in any organization or entity with any financial interest or non‐financial interest in the subject matter or materials discussed in this manuscript.

## DATA_AVAILABILITY STATEMENT

The data that support the findings of this study are openly available in [repository name e.g “figshare”] at http://doi.org/[doi], reference number [reference number].

## CONSENT

Written informed consent was obtained from the patient to publish this report in accordance with the journal's patient consent policy.
